# Mobile Health Information and Decision Support System Architecture: Enhancing Maternal Healthcare Access in Uganda

**DOI:** 10.24248/eahrj.v9i2.848

**Published:** 2025-12-24

**Authors:** Martin Morgan Kusasira, Anthony Atwiine, Joseph Wamema

**Affiliations:** a Uganda Christian University; b Uganda Martyrs University; c Makerere University

## Abstract

**Background:**

Maternal Health Information (MHI) is vital for empowering Pregnant and Postpartum Mothers to make informed decisions. However, access to this information remains a significant challenge in low-resource countries (LRCs) like Uganda, contributing to unacceptably high maternal mortality rates.

**Methods:**

This study adopted a qualitative case study research design. Data was collected from 50 purposively selected respondents, including Pregnant and Postpartum Mothers, medical workers, and Village Health Team (VHT) members, using semi-structured interviews and questionnaires at two health facilities in Uganda.

**Results:**

The study identified key impediments to Maternal Health Information access, including financial instability, inadequate spouse support, long waiting times at health facilities, the negative attitude of health workers, and language barriers. While health facilities were the primary source of information, Maternal Health Information was often reported as untimely and irrelevant to mothers’ specific needs.

**Conclusion:**

The proposed architecture is designed to bridge the gaps by extending relevant, reliable, and multilingual MHI directly to mothers, thus empowering their decision-making process and having the potential to reduce maternal mortality in Uganda and similar low resource countries.

## BACKGROUND

Maternal mortality remains a major global threat, with over 800 women dying every day from pregnancy and childbirth-related complications.^1–3^ 99% of these maternal deaths occur in Low Resource Countries (LRCs).^1–4^ Women often die or suffer debilitating diseases and injuries during pregnancy and childbirth due to postpartum haemorrhage, hypertensive disorders such as pre-eclampsia and eclampsia, infections, and abortions.^3^ Most maternal complications develop during pregnancy, while others pre-exist and worsen without timely and appropriate care.^5^ Strategies for reducing maternal mortality have been adopted globally, encompassing intrapartum care by skilled birth attendants, community health workers, traditional birth attendants, emergency obstetric care strategies, and antenatal and postpartum care, among others.^6,7^

mHealth (mobile health) is increasingly recognised as a “wonder drug” for rapidly transforming healthcare service delivery through diagnostic and treatment support, remote data collection and monitoring, disease prevention and epidemic outbreak tracking, cost reduction, and improvement of communication among health workers and patients.^8,9^ Thus, mHealth interventions have been widely regarded as practical and scalable solutions to improve maternal health.^10^ The WHO defines mHealth as a medical public health practice supported by mobile devices, including mobile phones, patient monitoring devices, wireless devices, and personal digital assistants.^11^ Today, mHealth applications rapidly transform healthcare service delivery globally.^12^ The potential for mHealth to impact global public health is enormous,^9^ and these applications have been widely acknowledged to transform the way health consumers and health providers exchange information.^13,14^

While High-Income Countries remain at the forefront of developing the latest mobile technologies used in healthcare (mHealth), the penetration rate of such technologies in low-resource countries has recently exceeded that of high-income countries.^15^ The increased cell phone penetration presents the potential for mHealth to improve preventive maternal healthcare services.^16^ As a result, various interventions in mHealth have been designed and implemented in several LRCs, including Uganda.^17,18^ Their impact has been shown to improve medication adherence in some patients, mobile data collection and communication tools for health workers,^19^ facilitate the attendance of skilled delivery and improve utilisation of maternal health (ANC/PNC) services.^20–22^

However, despite the reported positive outcomes, maternal mortality remains a significant public health threat in LRCs, including Uganda.^23^ Guille et al, ^24^ concluded that women in LRCs are not able to make informed choices and decisions to seek care because of limited recognition of danger signs, inadequate information about treatment options, associated harms and benefits, insufficient birth preparedness and implications of treatment decisions. Consequently, women fail to recognise a problem early enough to seek care, leading to maternal deaths. Several scholars, such as,^25–27Z^ have urged the need for women's awareness to empower their decision-making process regarding maternal and child health. Maternal mortality has been linked to women's healthcare decision-making power at the household level in many LRCs.^28^ Hossain and Hoque^29^ also noted that utilisation of maternal healthcare services is more common and frequent among women who are empowered in decision-making in matters related to healthcare. Hossain^30^ supported this, adding that such women can effectively utilise all available information and resources.

Maternal Health Information (MHI) is a fundamental building block of women's health during pregnancy, childbirth, and the postpartum period.^31^ The World Health Organization (WHO) defines Maternal Health Information as the facts related to women's health during pregnancy, childbirth, and postpartum.^32^ MHI is vital in supporting Pregnant and Postpartum Mothers to make informed decisions related to their health, improve self-care abilities, effectively interact with health providers, engage in preventive health behaviours, and utilise maternal health services.^33–35^ Access to relevant, timely and reliable Maternal Health Information is crucial in creating awareness and empowering women to make informed health decisions, practice self-care, and improve interaction with health providers.^33,36^ However, in many low-resource countries, including Uganda, many gaps prevail in access to quality information and decision support during the continuum of maternal care.

Despite the potential benefits of Maternal Health Information, Mothers in LRCs, including Uganda, face several barriers to accessing maternal health services, contributing to unexpectedly high maternal mortality.^34,35,37^ Previous studies on LMICs highlight long distances to health facilities, lack of decision-making power of women, language barrier, long waiting times at health facilities, negative attitude of health providers, feeling ashamed or embarrassed to talk about pregnancy-related issues, and cultural beliefs as some of the barriers.^26,34,37–41^

While numerous mHealth interventions have been implemented in low-resource countries such as RapidSMS-MCH in Rwanda, Wired Mothers in Zanzibar, and SMS-based reminder systems in Uganda, these operate in isolation, addressing only single facets of the broader challenge. Existing systems rely heavily on community health workers and offer limited direct empowerment for mothers themselves. Many mothers still lack adequate knowledge of birth preparedness, complications, and danger signs. Additional barriers, including financial instability, transport challenges, and limited partner support, further restrict timely care-seeking. Existing interventions, such as SMS or voicebased reminder systems, rely heavily on community health workers (CHWs) and do not directly engage mothers in the decision-making process. This creates delays in seeking care and reinforces dependency on intermediaries. Furthermore, current tools rarely integrate essential support services such as financial transactions for maternal needs, emergency transport, or interactive communication between mothers and healthcare providers during pregnancy and the postpartum period.

Therefore, this study specifically addresses the gap of absence of an integrated architectural framework that simultaneously tackles the multifaceted barriers to Maternal Health Information access, including financial constraint, transport challenges, limited partner involvement, and delayed decision making, within a unified mobile health system. To respond to this need, the study develops and proposes a Mobile Health Information and Decision Support System Architecture (MHIDSSA) designed to empower Pregnant and Postpartum Mothers directly through timely, relevant, reliable, and contextspecific information, enhanced decision-support tools, and improved access to maternal healthcare services.

## METHODOLOGY

### Research Design

This study adopted a case study design in which two health facilities, Masafu General Hospital in Busia District and Komamboga Health Centre III in Kampala District, served as the two distinct cases for in-depth investigation. These facilities were deliberately selected to represent contrasting geographical and contextual settings. Masafu General Hospital reflects a rural healthcare environment in Eastern Uganda, while Komamboga Health Centre III represents an urban setting in the capital city. This comparative approach enabled the study to capture the diversity of maternal health information access barriers and contextual challenges faced by pregnant and postpartum mothers across different health system contexts. The case study design was particularly appropriate for this research because it allowed for a comprehensive, context-rich exploration of real-life phenomena^42,^ specifically the barriers to maternal health information access and the design of a relevant mobile health information and decision support architecture. By examining both rural and urban cases, this study was able to triangulate findings, enhance the credibility and transferability of results, and then derive insights applicable to broader low-resource settings in Uganda. The two cases also facilitated identification of similarities and differences in awareness levels, transport accessibility, health worker interactions, and the financial challenges that influence maternal health decision making, which directly informed the proposed system architecture.

### Sampling Method

The study adopted purposive sampling, and participants were purposively selected from Pregnant and Postpartum mothers, Village Health Team members, and obstetricians. Personal judgment was used to choose members of the population to participate in the study and the cases that helped to answer research questions to achieve the research objectives.^44,45^ Purposeful sampling was used to select the case studies and the key respondents.

### Inclusion Criteria For Study Sites

This study was conducted at two health facilities in Uganda: Masafu General Hospital in Busia District and Komamboga Health Centre III in Kampala District. These health facilities were considered because they are under the Ministry of Health of Uganda, whose mission is to “provide the highest possible level of health services to all people in Uganda through the delivery of promotive, preventive, curative, palliative and rehabilitative health services at all levels”.^46^

Two case studies were adopted to gain deeper insights into differences in maternal health information access between rural and urban settings. Masafu General Hospital was specifically considered because it is in the eastern region of Uganda, which is the poorest in the country, with some of the worst MCH indicators and the highest maternal mortality rate.^47^ This study's sampling population included PPMs, VHTs, and Medical workers. The reason for adopting PPMs in this study was that they are directly subjected to maternal deaths. Therefore, the researcher expected them to provide information about their challenges in accessing MHI and services.

Conversely, the reason for adopting VHTs and Medical workers is that they are the health workers who attend to the mothers in the community (For VHTs) and at the health facilities (For Medical workers). Therefore, the researcher expected them to share their experiences, views, and opinions about these women's challenges and how the existing challenges can best be addressed. Based on these views, the study aimed to develop a solution to address the challenges.

### Data Collection Tools

Since the study sought several opinions, thoughts, attitudes, experiences, and views of respondents, semi-structured interviews and self-administered questionnaires were used. Data collection techniques, including semi-structured interviews, were used to collect qualitative data from medical workers and facility managers to get their thoughts and opinions on how best the issues raised by mothers could be addressed. Self-administered questionnaires were used to gather information from mothers about the challenges they face in accessing MHI for decision-making and how best they think these challenges could be addressed.

To ensure data quality, all data collectors underwent rigorous training on the study protocol, techniques for accurate and unbiased data collection, and ethical considerations. Data collection tools were pilot tested to identify and rectify any ambiguities or inconsistencies. The validated and standardized questionnaires and interview guides were used to ensure consistency. Field supervisors regularly monitored data collection activities and conducted random checks to ensure adherence to procedures. Completed data forms were reviewed daily for completeness and accuracy, and any discrepancies were promptly addressed. The study, where applicable, applied double data entry to detect and correct entry errors. To address literate versus illiterate participants, the study used trained interviewers, simplified language and visual aids, and voluntary participation and informed consent. To protect participants’ confidentiality and privacy, the study embraced anonymization, confidential interview settings, ethical approval, data use limitation, and secure data storage.

### Data Analysis

Data collected was analysed using thematic analysis, following the structured steps outlined by Coliazzi.^48^ This approach was selected because it provides a systematic framework for identifying, organizing, and interpreting recurring patterns and meaning within qualitative data. Although Colaizzi's method is commonly applied in phenomenological research, in this study, it was adopted to guide the thematic analysis process, ensuring a rigorous and transparent derivation of themes from participant narratives and questionnaire responses.

The analysis involved familiarization with data through repeated reading of transcripts, extraction of significant statements, formulation of meanings, clustering of related meanings into themes, and validation of the emergent themes against the raw data. This process enabled the identification of key barriers to maternal health information access, which directly informed the design of the proposed Mobile Health Information and Decision Support System Architecture (MHIDSSA).

### Ethical considerations

The study obtained research clearance from the necessary parties. Permission to carry out this research was granted in writing by the Directorate of Public Health and Environment of Kampala Capital City Authority and Masafu General Hospital (see reference letter DPHE/KCCA/1301), and all respondents consented to participate in the study.

## RESULTS

Table 1 summarizes respondents’ settings characteristics across the two case studies (Masafu General Hospital and Komamboga Health Centre III). The diversity of participants enhanced the study's ability to identify crosscontextual barriers in both the rural and urban settings, thus addressing Objective 1 of the study.

**TABLE 1:** Characteristics of Respondents (N=50)

Respondent Categories	Rural Category code	Urban Category code	Number of respondents	Respondents’ percentage
Pregnant mothers	R	KR	20	40%
Medical Workers	MHW	KHW	12	24%
Village Health Team (VHT) Members.	VHTM	KVHT	8	16%
Postpartum Mothers	MPP	KPP	10	20%
Total			50	100%

The study utilized a total of 50 respondents (n=50) under the categories of twenty (20) pregnant mothers (10 from a rural setting and ten (10) from an urban setting), ten (10) postpartum mothers of at most 42 days after delivery (five (5)from a rural location and five (5) from urban setting), twelve (12) Medical workers (6 from an urban environment and six (6) from a rural setting) and eight (8) VHT members four (4) from rural area and four (4) others from urban area). These participants were purposefully chosen based on the anticipation that they have good knowledge about information related to pregnancy, childbirth, and the postpartum period. Additionally, these respondents were thought to thoroughly comprehend the challenges encountered in accessing MHI.

The results in this study are centered on 50 responses acquired from pregnant mothers, who composed forty per cent (40%) of the respondents, making it the highest percentage. The Medical workers followed with twenty-four per cent (24%), Postpartum mothers twenty per cent (20%), and VHTs sixteen per cent (16%) performing in the field of Maternal and Child Health at the village level.

The findings are presented in the following sub-sections of seven significant recurring themes: Awareness of MHI services, distance to health facilities, long waiting times, financial instability, spouse support, negative attitude of health workers, and language barrier.

### Awareness of Maternal Health Information (MHI) Services

**FIGURE 1:**
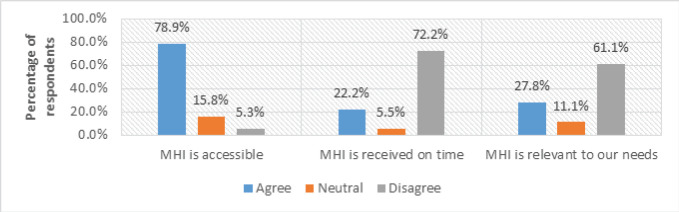
Respondents’ Awareness of Maternal Health Information Services

**FIGURE 2:**
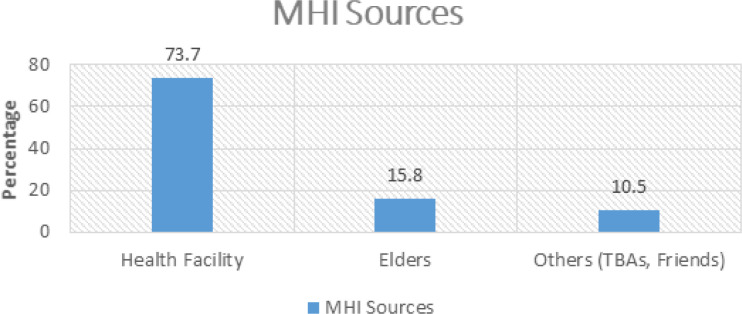
Sources of MHI

Figure 1 below shows respondents’ views regarding Maternal Health Information Services (MHIS) awareness. The responses are linked to whether Maternal Health Information is available for mothers, and different attributes were assessed, including accessibility, timeliness, and relevance to the needs of pregnant and postpartum mothers.

(66.7%, n=20) of respondents from both rural and urban areas agreed that they are aware of the availability of maternal health information services, while (22.2%, n=6) disagreed. The respondents (66.7%, n=20) who reported the availability of MHIS were further probed to determine where they obtained this information. Figure 2, below, represents the respondents’ views, showing that (73.7%, n=15) of respondents get MHI from Health Facilities, (15.8%, n=3) from Elders, and (10.5%, n=2) from other sources like Traditional Birth Attendants (TBAs) and Friends.

This part of the study emphasised mothers whose source of maternal health information is health facilities. Therefore, the mothers (73.7%, n=15) who reported health facilities as their MHI source were asked if this information was accessible (see Figure 1). (78.9%, n=12) of them reported that the information is easily accessible. The researcher further asked this percentage of respondents to determine if they accessed maternal health information on time. (72.2%, n=11) said that they do not have access to MHI on time. The majority complained that they take longer to receive maternal health information that could help them make decisions about seeking maternal health care and for informed health decision-making, as commented by this respondent, who said:

*“Today is not my clinic day, but I came to consult the health workers because I am not feeling well. Last night, I developed a fever plus flu and sought help from the health workers. When I gave my chart to the health worker, she recommended me to another, saying she couldn't help me because her duties were specific to a different category of patients.” – (Interview with KR5 on 12*^*th*^ *October 2021*, Komamboga HCIII).

Additionally, the respondents (78.9%, n=12) who said they have access to MHI were further asked if this information is relevant to their needs. (61.1%, n=7) of respondents disagreed, reporting that the information they have access to is irrelevant and does not satisfy their maternal needs, as evidenced in this interview response:

*“When I come to the facility, health workers talk to us about some pregnancy-related information, but much remains untold. For example, I would wish to know how best I should take care of myself and my child to avoid complications like the miscarriage that I recently had.” – (Interview with MPP1 on 27*^*th*^ *September 2021*, Masafu General Hospital).

Mothers, especially in Masafu General Hospital (rural case), reported that they primarily depend on verbal information from village health teams (VHTs) and occasional antenatal sessions. In contrast, mothers from Komamboga HCIII (urban case) mentioned exposure to health campaigns and posters yet still lacked consistent access to personalized maternal health information.

These findings address objective 1, which sought to identify the challenges pregnant and postpartum mothers face in accessing MHI. The results show that low awareness limits the ability of mothers to make informed health decisions and delays the recognition of danger signs during pregnancy and childbirth.

The observed low awareness highlights the critical need for a centralized and accessible information platform where mothers can receive verified, context-specific health information directly. Accordingly, the proposed Mobile Health Information and Decision Support System Architecture (MHIDSSA) include an information access module designed to bridge this awareness gap by enabling the timely dissemination of maternal health messages through mobile and multimedia interfaces.

### Distance to Health Facilities

**FIGURE 3:**
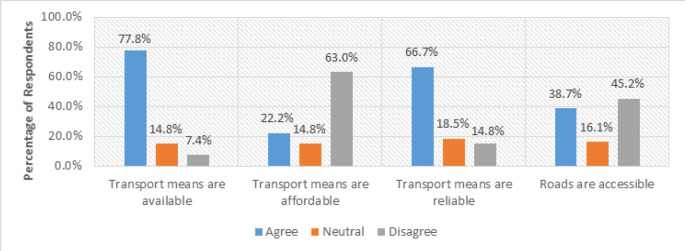
Views on Transport and Road Accessibility (N=15)

Relying on the results of this study, which revealed the primary source of MHI as health facilities (73.7%, n=15 (Figure 2), distance to health facilities was probed to gain an insight into the means of transport used by mothers to access health facilities, accessibility of roads, availability, affordability, and reliability of transportation means as shown in Figure 3.

Regarding the means of transport, (74.1%, n=11) of respondents revealed that they use motor bicycles, locally known as *boda-boda;* (18.5%, n=3) walked to reach the facilities, while (7.4%, n=1) said they use bicycles. The (18.5 %, n=2) of respondents who said they walk could have represented those who reside near the health facilities where they access MHI and those who cannot afford public transport. Regarding road accessibility, respondents were asked whether the roads are accessible. (45.2%, n=6) of them disagreed, reporting that the roads are not easily accessible, as commented by a respondent who said:

*“Most mothers in Bukobe Parish deliver from home because the place is too far from Masafu, and the road is too bad to access this place, yet we don't have a Health Centre Two in Masafu.” – (Interview held with MHW1 on 27*^*th*^ *September 2021*, Masafu General Hospital).

Furthermore, the respondents (81.5%, n=12) who use both bicycles and motor bicycles) were inquired about these means of transport availability. (84.6%, n=10) of the respondents agreed that the transport means are available. The respondents (84.6%, n=10) who confirmed the availability of transport means were further queried about its reliability. (66.7%, n=10) of them agreed that the means are reliable. However, some respondents complained about unreliability, as evidenced by a respondent who said:

*“Many times, you call a boda-boda man to come for you from deep down in the village, and they fail to come or take long to come. When they arrive, they must drive carefully so as not to cause any injuries because one is pregnant. By the time you reach, the health talk is done.” – (Interview with MPP3 on 27*^*th*^ *September 2021*, Masafu General Hospital).

The respondents (84.6%, n=10) who said that the transport means are available were also asked about affordability. Only (22.2%, n=2) of them agreed that the means are affordable. However, (63%, n=6) of the respondents disagreed, as revealed by a respondent who said:

*“It is not affordable to always come to the health facility as health workers require. It is costly in terms of transport”. – (Interview with KR3 on 12*^*th*^ *October 2021*, Komamboga HCIII).

The study notes that mothers lived far from the health facility, making it difficult for them to arrive in time for antenatal and postnatal sessions where important maternal health information is shared. This challenge was more pronounced in the rural case of Masafu General Hospital, where long walking distances and poor road conditions often discourage mothers from regular health facility visits. In contrast, respondents from Komamboga HCIII (urban case) experienced shorter physical distances but still cited transport costs 63% (n=9) as a significant challenge.

The findings directly contribute to Objective 1 of the study, which aimed to identify the challenges faced by pregnant and postpartum mothers in accessing MHI. The contrast between rural and urban experiences highlights the need for innovative solutions that can minimise travel barriers. Consequently, the proposed Mobile Health Information and Decision Support System Architecture (MHIDSSA) integrates a Transport Coordination Module designed to connect mothers with local transport providers and community health facilities, thereby reducing delays and improving timely access to maternal health information services.

### Long Waiting Time at Health Facilities

**FIGURE 4:**
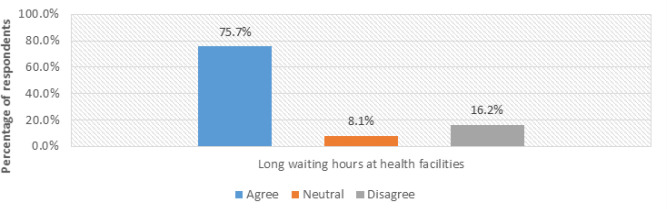
Respondents’ Views on Waiting Times at Health Facilities (N = 30)

As represented in Figure 4, majority of respondents reported that long waiting hours at health facilities discouraged them from attending maternal health information (MHI) sessions.

Out of the 50 respondents (75.7%, n=38) agreed that the long waits exist and affect timely access to MHI, as evidenced by an interview response:

*“The long waits in queues force mothers to come once for only the first ANC visit and then return during labour. This makes it difficult for health workers to provide information about their pregnancies and identify early complications.” – (Interview with VHTM2 on 27th September 2021*, Masafu General Hospital).

**FIGURE 5:**
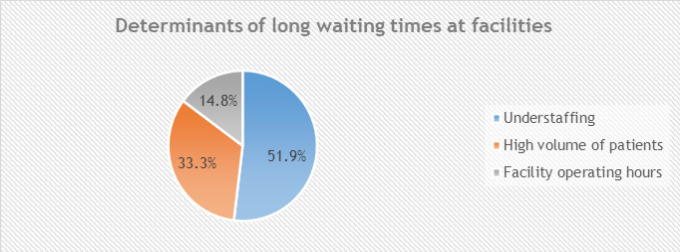
Determinants of Long Waits

The study further delved deep to investigating the causes of these long waits at the facilities during MHI access to gain insight into the challenge of long waiting, as indicated in Figure 5.

As illustrated in Figure 5, (51.9%, n=26) of respondents reported that the long waits at the facilities were attributed to understaffing, (33.3%, n=16) to the high volume of patients, and (14.8%, n=7) to the facility operating hours. The problem was more severe in Komamboga HCIII (urban case), where high patient volumes and limited staffing led to congestion and prolonged queues. Mothers in Masafu General Hospital (rural case) also noted delays, often due to the small number of attending midwives and unpredictable health-worker availability. One respondent had this to say:

*“We have been working on eighty mothers, only two health workers since morning. There is a series of activities to take mothers through, from the health talk to clerking, weight taking, palpation, ambulation, tests, diagnosis, and prescription after tests. It is a lot of work for only two health workers.” – (Interview with MHW2 on 28*^*th*^ *September 2021*, Masafu General Hospital).

These results directly address Objective 1 of this study, which sought to identify challenges faced by pregnant and postpartum mothers in accessing MHI. The findings reveal that long waiting times not only cause fatigue and frustration among mothers but also reduce participation in health education sessions, leading to missed opportunities for critical health information.

To address this issue, the proposed Mobile Health Information and Decision Support System Architecture (MHIDSSA) incorporates a Communication Module, which allows mothers to receive timely updates, reminders, and notifications from health facilities, thereby minimizing overcrowding and ensuring that health information sessions are delivered to mothers even when they cannot physically wait for long periods.

### Financial Instability

The study investigated the sources of income of PPMs, how reliable these sources are, and their sufficiency to perceive the impact of financial instability on MHI access.

**FIGURE 6:**
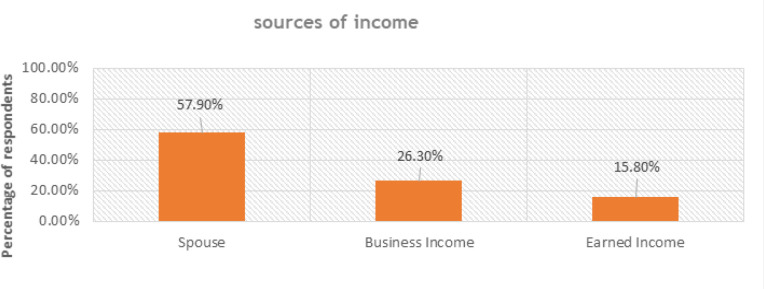
Sources of Income for pregnant and postpartum mothers (N=30)

As presented in Figure 6, (57.9%, n=17) of mothers reported getting financial support from their husbands, (26.3%, n=8) said they have small businesses, and (15.8% n=5) also revealed that they earn income from employers.

The respondents were further asked to find out whether these sources of income are reliable. (63.2%, n=19) of the respondents disagreed, as indicated by a respondent in an interview:

*“This year, I developed some continuous abdominal pain, and I asked my husband to give me money to go to the hospital to seek advice about it, but he kept telling me that he had no money until I miscarried.” – (Interview with R5 on 27*^*th*^ *September 2021*, Masafu General Hospital).

Similarly, the 30 pregnant and postpartum mothers were asked to learn whether their incomes were adequate or enough to enable them to access MHI when required, and the majority (78.9%, n=23) differed from this, as exhibited in the interview response of this respondent:

*“My husband cannot fully support me financially because he is still a youth and has no stable income. Many times, it is my parents who come in to support me and my child. That's how it was even during pregnancy.” – (Interview with MPP2 on 27*^*th*^ *September 2021*, Masafu General Hospital).

**FIGURE 7:**
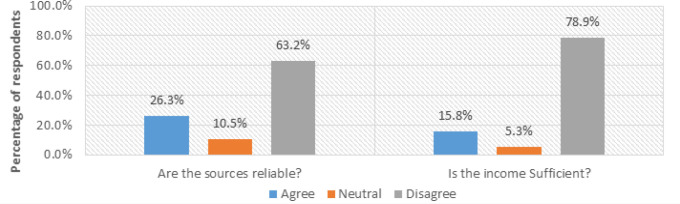
Respondent views on financial instability (N=30)

This study revealed that irregular or insufficient income constrained the mothers’ ability to travel to health facilities, buy communication airtime, or meet their day-to-day needs during pregnancy and postpartum. Participants from Masafu General Hospital (rural case) explained that most households depend on subsistence farming and informal work, which provide little or unsteady income. On the contrary, mothers from Komamboga health centre III (urban case) engaged in some small-scale trading and casual employment yet still struggling with high costs of living and competing household responsibilities. (Figure 7)

These findings directly contribute to Objective 1, which sought to identify challenges faced by pregnant and postpartum mothers in accessing MHI. They demonstrate how limited and unstable income affects both the affordability and priority of health-seeking behaviour.

The analysis shows that even in the face of free maternal health services, hidden costs such as transport, meals, and communication expenses significantly restrict access to information and timely care. To address this challenge, the proposed Mobile Health Information and Decision Support System Architecture (MHIDSSA) incorporates the transaction Module (MamaSente) to support financial support groups, and access to emergency assistance, thus reducing financial barriers to health information and services access and utilization.

### Spouse Support

**FIGURE 8:**
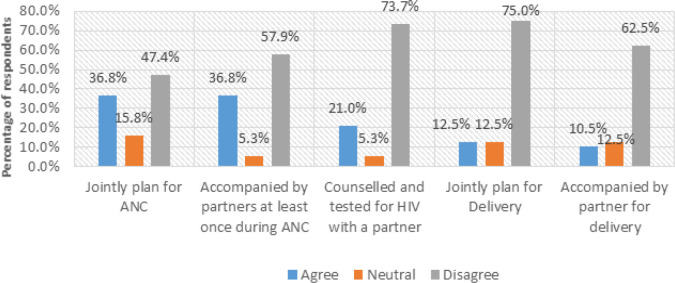
Respondents’ Views on Spouse Support (N=30)

The study also investigated how spouse support affects access to MHI during pregnancy and childbirth, as illustrated in Figure 8, respondents were invited to give their views on whether they jointly plan for ANC with their spouses, accompanied by partners during ANC visits at least once, counselled, and tested for HIV with a partner, accompanied by the partner for delivery and perhaps planned for delivery with a partner.

Out of the 30 pregnant and postpartum mothers, only (36.8%, n=11) of mothers agreed that their spouses are involved in planning for ANC, while (47.4%, n=14) disagreed. (57.9%, n=17) disagreed with being accompanied by their partners at least once during ANC, and (73.7%, n=22) revealed that their partners have never been present with them for counselling and HIV testing. Regarding jointly planning for delivery, (75.0%, n=22) of mothers disclosed that their partners don't jointly arrange for delivery, and (62.5%, n=18) expressed that their spouses have never accompanied them. Some respondents who revealed that their partners have never accompanied them to any ANC visit (57.9%, n=17) had this to say:

*“When you ask him to come to the health facility as instructed by health workers, his reply will be, ‘ Am I going to give birth too? Men are unsupportive. – (Interview with KR4 on 12*^*th*^ *October 2021*, Komamboga HCIII).

On the issue of mothers being accompanied by their partners for delivery, (62.5%, n=18) of them disagreed.

The limited partner involvement was more common in participants from the rural case (Masafu General Hospital), where cultural norms often define pregnancy and childbirth as women's affairs. Respondents from Komamboga HCIII (urban case) indicated a slightly higher level of spouse support and partner involvement, but still described minimal participation in attending antenatal and postnatal visits or decision making regarding maternal healthcare.

These findings directly relate to Objective 1, which sought to identify challenges faced by pregnant and postpartum mothers in accessing MHI. The limited male involvement contributes to poor maternal health outcomes and missed opportunities for shared learning. To address this gap, the proposed Mobile Health Information and Decision Support System Architecture (MHIDSSA) integrates a Communication Module designed to facilitate direct engagement between mothers, spouses, and their healthcare providers. This module allows partners to receive tailored maternal health updates, appointment reminders, and educational messages, promoting male involvement in maternal health decisions and enhancing support for mothers.

**FIGURE 9:**
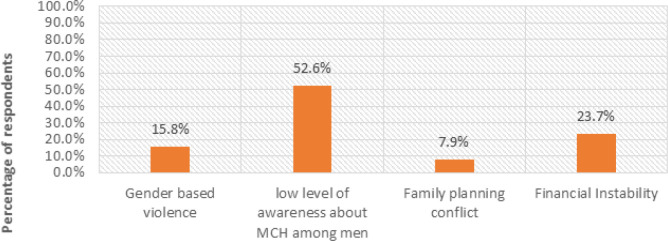
Causes of inadequate partner support (N=50)

After gaining a deep insight into partners’ moral, financial, and social support, this study also investigated the factors attributed to inadequate partner support during pregnancy and postpartum, as illustrated in Figure 9.

The study's findings indicated that (15.8%, n=8) of the respondents’ views were tailored to Gender-Based Violence (GBV), (52.6%, n=26) were related to a low level of awareness about MCH among men, (9%, n=4) were related to family planning, while financial instability covered (23.7%, n=12). Below is an expression associated with GBV.

*“Many men in this region force women to go and abort, saying that they are not ready for the child. And when the mother refuses, she goes through this alone until delivery”. (Interview held with KVHT1on 13*^*th*^ *October 2021*, Komamboga HCIII)”

### Health Workers’ Negative Attitude

**FIGURE 10:**
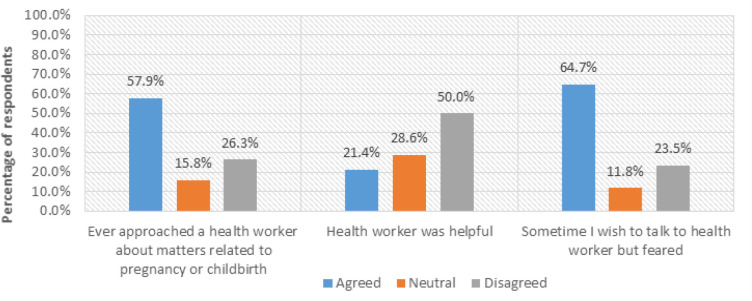
Views on Health Workers’ Attitude (n=30)

Figure 10 shows respondents’ views regarding health workers’ negative attitudes towards PPMs. Respondents were asked whether they had ever approached a health worker about matters related to pregnancy, whether the health worker was helpful, and whether there was a time when they wished to seek advice from a health worker but feared, and what caused that fear.

Regarding whether respondents’ mothers ever approached a health worker about pregnancy or childbirth, (57.9%, n=17) agreed, while (26.3%, n=7) disagreed. The respondents (57.9%, n=17) who said they had ever approached a health worker on pregnancy and childbirth-related issues were further asked whether the health workers were helpful. Only (21.4%, n=3) of respondents agreed. However, (50.0%, n=8) of respondents disagreed, as reported by a respondent who said:

*“I had a miscarriage last year, and when I approached a health worker to explain what caused it, she didn't tell me. A few weeks ago, I started getting the same symptoms that preceded the other miscarriage. When I consulted another health worker about the same issue, she wrote down something and asked me to take a test”. – (Interview held with R9 on 27*^*th*^ *September 2021*, Masafu General Hospital).

**FIGURE 11:**
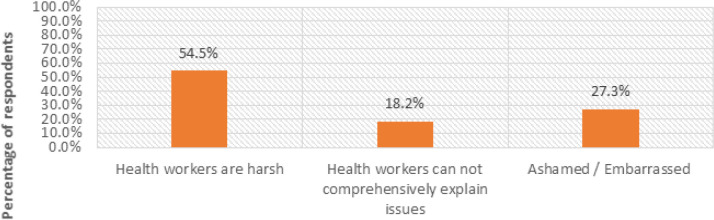
Reasons for Clients’ Fears to Approach Health Workers

The mothers were also asked whether there was a time when they wanted to approach a health worker about matters related to pregnancy and childbirth, but feared. (64.7%, n=19) of them agreed, while (23.5%, n=7) disagreed. To gain a deeper understanding of the matter, the respondents (64.7%, n=19) who said that they had ever desired to seek maternal health advice from health workers but feared were further asked why they feared approaching the health workers. The views in Figure 11.

Attitude of health workers was frequently cited as a key factor in influencing mothers’ willingness and ability to seek maternal health information (MHI). Respondents from Masafu General Hospital (rural case) described instances of being scolded or ignored, particularly when they arrived late or without essential materials. In Komamboga HCIII (urban case), some participants acknowledged more supportive interactions but still reported occasional impatience and poor communication during busy clinic hours.

These findings address Objective 1, which sought to identify challenges faced by pregnant and postpartum mothers in accessing MHI. Negative attitude of health workers not only discourages health literacy session attendance but also limits the quality of information exchanged between the mothers and healthcare providers. To mitigate this, the proposed Mobile Health Information and Decision Support System Architecture (MHIDSSA) integrates an Interactive Communication Module that promotes continuous engagement between mothers and health workers. Through this module, mothers can access verified health information and also send inquires without fear of judgement. In addition, health workers can also disseminate guidance through friendly digital channels, thereby fostering trust and improving the maternal health communication environment.

### Language Barrier

To have an insight into whether PPMs understand the language in which they receive MHI, this study also considered the opinions and views of participants about the matter. When asked whether they correctly understand the language in which they receive MHI, the Majority (52.6%, n=15) of the mothers reported that occasionally they fail to comprehensively understand what the health workers tell them during the dissemination of MHI, especially in the form of written material, often delivered in the English language or technical medical terms. Similarly, out of the 20 health workers whose opinions were sought for whether they think mothers correctly understand the MHI provided to them, (63.2%, n=12) revealed that the language barrier is still an issue in the dissemination of MHI, as exhibited in the response below:

*“The urban setting houses many people who speak different languages. We even get many mothers who understand neither Luganda nor English.” – (Interview with KHW1 on 13th October 2021*, Komamboga HCIII).

Language barrier was more pronounced among mothers from Masafu General Hospital (rural case), where most respondents preferred using local languages such as Samia and Lugwere. At Komamboga HCIII (urban case), respondents expressed slightly better understanding levels due to higher literacy level rates, but still indicated the need to translate maternal health literature to local languages, especially Luganda, for better comprehension.

Health workers also acknowledge that language differences limit effective communication and participation during health education sessions. The lack of localized communication tools restricts message delivery to low-level literate mothers, especially in rural settings. To overcome this limitation, the proposed Mobile Health Information and Decision Support System Architecture (MHIDSSA) incorporates a multi-lingual and multimedia Module that provides maternal health information through translated text, audio, and video content in commonly spoken languages. This module bridges the gap between the literate and non-literate mothers, enabling them to access and understand vital maternal health information with convenience.

The above impediments formed a clear basis for the requirements that guided the design of the Mobile Health Information and Decision Support System Architecture (MHIDSSA) aimed at improving MHI access in Uganda. The themes identified from the study collectively revealed the multifaceted nature of barriers to timely and reliable MHI access. (Table 2)

**TABLE 2:** Proposed Mobile Health Information and Decision Support System Architecture

Challenges	Requirements
Low level of awareness about MCH services	Interactive MCH Information Hub
Limited access to relevant and timely MHI	Real-time access to MHI through SMS reminders, the Interactive Voice Response Platform, and the Emergency Centre
Unaffordable transport means	Instant access to affordable Transport through the GPS-supported Mama Boda app, integrated with the Mama Sente
Financial Instability	Maternity saving scheme / Mama Sente
Ignorance in men / a big knowledge gap in men about MCH.	MCH programme for men / Men involvement platform
Language barrier	Multilingual Information platform for mothers
Harsh health workers during information provision.	Provision of downloadable material, audio material, and access to available professionals for inquiries

### Architecture principles

Architecture principles are a set of general rules and guidelines that relate to architecture work and the basis for architecture development.^49^ The following are the goals and principles that guided the design and implementation of the Mobile Health Information and Decision Support System Architecture for improving access to MHI in Uganda. Table 3.

**TABLE 3:** Adopted Principles for Development of System Architecture

Principle	Statement	Rationale	Implication
Business Continuity	Despite system interruptions, enterprise operations are maintained. In other words, Hardware failure, data corruption and natural disasters shouldn't disrupt the enterprise's activities.	The more system operations become extensive, the more we depend on them. Therefore, this principle will be highly considered to promote system reliability throughout the design, maintenance, and use of this application.	This projected the ability of system functions to continue operating on alternative information delivery mechanisms like SMS platforms to support MHI access as an alternative to the web.
Data as an Asset	Data is an asset that has value to the enterprise and is managed accordingly.	All decisions of pregnant and postpartum mothers are made based on data provided during pregnancy and childbirth. Therefore, using this principle, data will be organised and managed carefully to ensure that it is accurate, reliable, & timely available when needed.	The MHI accessed by mothers is provided by licensed regional senior medical workers and nutritionists verified by the Ministry of Health. The MHI management module manages all data in the application to ensure data quality (accuracy, completeness, relevance, timeliness, and reliability).
Data is shared	Data should be stored within one application & shared across the entire enterprise.	This principle is essential so everyone within the enterprise can access the data they need to do their job.	The Data Capture and Update API and the Transaction Management APIs are software intermediaries in the MamaApp that permit the extraction and sharing of data between the six modules that generate services, which services are utilised by the application functions to satisfy users’ needs.
Data is Accessible	Data is accessible for users to perform their functions.	Broad access to MHI will empower PPMs to effectively & efficiently make decisions related to their health during pregnancy and childbirth, thus promoting a timely response to service delivery & information requests.	MamaApp embraces the aspects of accessibility, such as multilingualism (information provided to PPMs is in different local languages), metadata (description & explanation of provided data in the application), & interoperability (MamaApp shares data across multiple devices, platforms, & payment mechanisms).
Data Security	Data is protected from unauthorised use and disclosure.	Open information sharing & the information release via relevant legislation will be balanced against the need to restrict the availability of classified, proprietary, & sensitive information.	The application adopted different methods of data protection at different levels These include Encryption, Authentication, and Authorization, Access control (passwords, multi-factor authentication, and role-based access control), inserting secure APIs (to ensure client data security within the mobile application), and backup \ and recovery.
Ease-of-Use	Applications are easy to use. The underlying technology is transparent to users, so they can concentrate on their tasks.	This principle will ensure that users spend less time figuring out how to use the application, which leads to less productivity and triggers delays in decision-making, but rather concentrate on performing required tasks. This is because users like mothers are not very educated. Therefore, they should find it easy to use the application.	The MamaApp adopted a minimalistic design approach to create a modest and highly usable mobile application. The strategy involves minimum actions, minimum cognitive load, maximised usability, simple actions and design, and all actions’ logic. These favour users to obtain information quickly and easily.
Interoperability	Software and hardware should conform to standards that promote data, applications, & technology interoperability.	The principle fosters the application's ability to provide data, information, & services to, and accept the same from, other applications or systems and to use the exchanged data, information and services for effective operation	MamaApp shares data across multiple devices, platforms, and payment mechanisms.
Requirements-Based Change	Only in response to business needs are changes to applications and technology made.	This principle will foster an environment where the information environment changes in response to the user's needs rather than making changes base on new technology.	Changes to this application should be based on the user's requirements and adequately documented.

### The Integrated Mobile Health Information and Decision Support System Architecture

**FIGURE 12:**
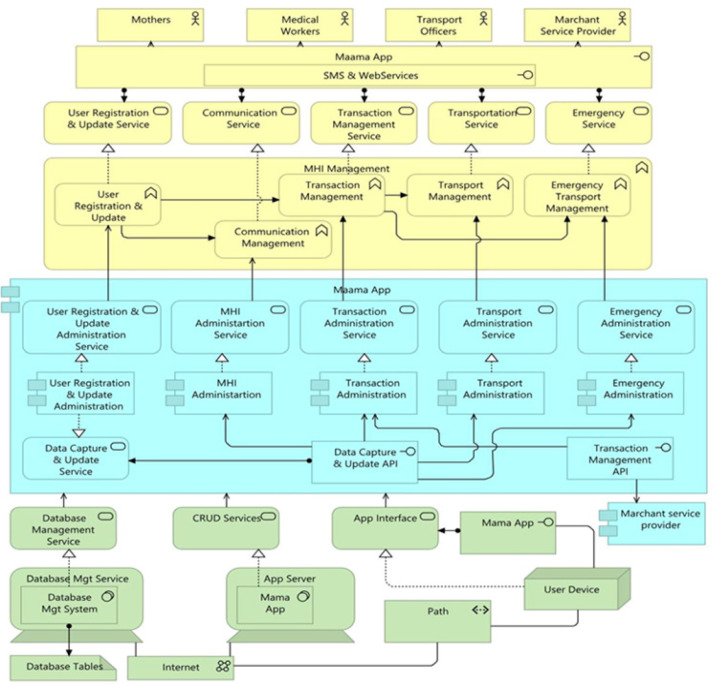
The Integrated System Architecture for Improving Maternal Health Information Access in Low Resource Settings

The application consists of five core functions. These are logical application components and logical units of the application capability, which require physical and organisational resources to be implemented. These include the user registration and update function, communication management function, transaction management function, transport management function, and emergency management function. The *User Registration and Update Function* is a core fundamental component in the proposed Mobile Health Information and Decision Support System Architecture (MHIDSSA). Although not explicitly revealed as one of the thematic results (since it's more structural than behavioral), it coordinates all tasks that enable the actors (Pregnant and Postpartum Mothers, Medical workers, Transport Officers, and Merchant Service Providers) of the application to create a new user account as a description of proving identity. It also allows modifying a registered user's profile, attributes, or events in the application. Its inclusion was a direct response to findings about awareness data management and follow-up challenges, particularly the difficulty health workers faced in identifying and continuously tracking pregnant and postpartum mothers, and managing maternal health information identified across both case studies. During data collection, Health workers reported difficulties maintaining accurate records of pregnant and postpartum mothers, while mothers themselves expressed frustration about having to repeatedly provide personal details during each visit. This applied to both the rural and urban case studies. This function directly responds to the awareness and follow-up challenges observed in 66.7% of respondents (section 3.1) who lacked awareness of available maternal health information services, indicating irregular contact with health facilities, thus a lack of continuity in information delivery. The proposed system architecture permits automatic status updates, for example, when a mother transits from pregnancy to postpartum, so that information remains relevant to her current stage. Figure 12

The *Communication Management Function* coordinates all tasks related to keeping mothers updated with multilingual multimedia MHI in images and graphics, text, video, and audio formats to inform and motivate mothers to make better decisions. Long waiting times were reported by 75.7% (n=38) of respondents, causing missed or shortened maternal health education sessions. The Communication Management Function was therefore introduced to streamline scheduling and provide real-time communication between mothers and healthcare providers. This module allows mothers to receive automated appointment reminders, queue updates, and rescheduling options, thus reducing congestion and saving time.

The *Transaction Management Function* controls the coordination of transactions over different resources. It is important to observe that financial instability emerged as one of the strongest impediments to maternal health information access, reported by 78.9% (n=23) of respondents (see section 3.4). This function was designed to support financial operations for MamaSente, and all payments for maternal-related services, thus reducing financial constraints that hinder access to information and care.

The *Transportation Management Function* controls the transportation activities of the application. It directly addresses the challenge of unaffordable and unreliable transport, reported by 63% and 66.7% of respondents respectively (Section 3.2). Respondents, especially from rural areas, described walking long distances and poor road infrastructure as major barriers to accessing care. This function links mothers with registered local transport providers and community transport officers, thus facilitating timely travel to health facilities, especially in emergencies.

The *Emergency Management Function* coordinates all the emergency activities that are performed by the application. This Function was incorporated to directly address the current “three delays” principle and access challenges faced by mothers during obstetric emergencies. Findings from the study showed that 66.7% of respondents reported difficulties obtaining timely assistance during maternal emergencies, largely due to unreliability of transport means, long distances and poor communication, and limited transport options (sections 3.1 and 3.2). Respondents from Masafu General Hospital described situations where mothers have to wait for neighbours or the Village Health Teams to find transport and occasionally wait for transport officers for long hours to pick them up for antenatal or postnatal visits, while those from Komamboga HCIII mentioned unstable and high transport costs during emergencies. This Component integrates real-time alerts and response mechanisms, allowing mothers or caregivers to send emergency requests directly to the nearest transport provider, healthcare worker or community responder. All these functions are bound by the respective services that they offer. Services with explicitly defined boundaries support the functions, and the services are realised and supported by business processes. The services supported by these functions include user registration and update service, communication service, transaction management service, transport service, and emergency service. These services provide materials and valuable information and real-time feedback to those who request them, and users access them through the MamaApp interface, which is supported by SMS and the Web.

To support the execution of the business functions, MamaApp has one external and five internal modules. These components are fundamental to the architecture, represent a single responsibility within the application, and communicate with the application via explicitly defined entry points. The modules represent an encapsulation of application functionality aligned to the implementation structure, and they are assigned to one or more application functions. The internal modules include user registration and update administration, MHI administration, transaction administration, transport administration, and emergency administration. The external module is the mobile service provider module. The *user registration and update administration* module encapsulates all the data and behaviours of user registration and updates into one logical unit, exposes the user registration and update service, and makes it available through the MamaApp interface. The *MHI administration* module represents the MHI application tasks and encapsulates the data model and activities associated with MHI gathering, processing, and dissemination. This module realises the MHI management service utilised by the Communication management function. The *Transaction administration module* captures application functionalities aligned to the sequence of actions between the application and other applications, and, in this context, the *Merchant service provider module*.

The merchant service provider module is an external application component that serves the *Transaction administration* application module with services accessed through the *Transaction Management API* to effectively realise the Transaction administration service, which is utilised by the *Transaction management function*. The *Transport Administration* module is a self-contained unit of functionality that controls activities provided by the Transportation Administration service, accessed by users through MamaApp. Finally, the *Emergency administration* module is an application component that captures all tasks tailored to urgent, unexpected situations that pose an instant risk to the health and life of mothers during pregnancy and childbirth, and require immediate action. The component discloses the emergency administration service utilised by the *emergency management function*. The different application modules provide each other with services through *Data Capture and Update.*

An infrastructure platform supports the entire Mobile Health Information and Decision Support Application. The platform comprises hardware and virtualised platforms that run services, applications, and components. It contains application servers and database servers. Application servers are network computers that store, run, and host applications for client computers. These reside on the server side, providing logic behind any application. The application server hosts the MamaApp, which realizes the CRUD (Create, Read, Update, and Delete) services. The CRUD services are fundamental operations for managing and manipulating data in applications and databases. These permit users to create, read, update, and delete data, thus forming a backbone of user and data interaction with persistent data. The database servers are networked computers on a standard network dedicated to storing and retrieving data for authorised users. The Database Management Server in the infrastructure layer above realises the Database Management Service, which is an integrated console for cloud and on-premises databases with lifecycle database management capabilities for monitoring, optimisation, performance management, and administration. Both servers are linked by a network (internet) connecting all devices and servers.

## DISCUSSION

This study examined the multiple, interconnected barriers that limit access to Maternal Health Information (MHI) among Pregnant and Postpartum Mothers in low-resource settings and used these findings to justify the development of an integrated Mobile Health Information and Decision Support System Architecture (MHIDSSA). The results indicate that informational, financial, transport-related, and social barriers reinforce one another and collectively hinder mothers’ ability to make timely and informed health decisions. This interdependence illustrates why fragmented interventions are insufficient and why a unified, multi-component system is required for meaningful improvement.

The respondents perceived several factors as impediments to maternal health information access, and these are particularly significant in clarifying the limited access to MHI. This study revealed that pregnant and postpartum mothers were aware of maternal health information services, and 78.9% (section 3.1) had access to this information. However, health workers did not timely avail this information, which was often irrelevant to maternal needs, thus limiting mothers in using maternal health information to make decisions concerning their health during pregnancy and childbirth. This finding concurs with previous scholars who disclosed that access to relevant and reliable MHI is crucial in creating awareness and empowering women to make informed decisions about their reproductive health.^33,36^ Providing adequate and relevant information is the first and most crucial step in helping pregnant women make informed decisions, and the quality of decisions made largely depends on the type of information available to them.^41,50^

According to this study, boda-bodas emerged as the prominent means of transportation, represented by 74.1% of respondents (section 3.2). 18.5% of them access facilities on foot, while 7.4% use bicycles. The 7.4% of respondents who said that they walk could have represented those who reside near the health facilities where they access MHI, and those who cannot afford public transport. Mothers disclosed that the means of transport are available (84.6%, section 3.2) and reliable (66.7%, section 3.2), but the majority cannot afford them. Also, mothers are threatened by the inaccessible roads in some regions that endanger their lives while finding their way to health facilities. In such situations, some mothers choose to walk to health facilities or stay at home rather than putting their lives and the lives of the neonates at risk. The findings from this study reflect previous results revealing that walking, bicycles, and motorcycles are the predominant means of transportation used by PPMs in rural and urban LRCs to access maternal health facilities.^51^ Kassim ^37^ also noted that because of poor road infrastructure, the only means of transport affordable to most women and their families is the motorcycle, locally referred to as ‘boda-boda’. Women walk long distances to access the road network to get transportation, but many need help to afford public transport, especially for routine visits to health facilities.^52^ Therefore, the long distance to health facilities is still a big issue to resolve to enhance access to MHI and service delivery in Uganda.

The critical factor in public transport unaffordability could be explained by the financial instability among pregnant and postpartum mothers, represented by insufficient income and unreliable income sources. The sources of income of mothers were primarily earned income (15.8%), business income (26.3%), and spouse (57.9%). The finding revealed that most mothers depend on their spouses to support their pregnancy and the postpartum period. However, this income needs to be more reliable. This indicates that at many stages in this period, PPMs lack support for maternal needs, especially maternal health information services, including information to help them make decisions related to their health.

Additionally, this study identified that men's awareness of MCH is still low. Banik^53^ indicated that Pregnant women in LRCs have been discouraged by the costs attributed to seeking maternal health services, hence failing to receive these services at the right time. This finding was supported by scholars who noted that women fall victim to high costs of MHI in the form of transport costs to health facilities to get information and attendance costs, among others, because most lack disposable income.^34,40^ They use the little they have to support themselves on food and clothing, leaving out information related to pregnancy and childbirth, putting their life and the children in danger.^31^

Moreover, men still need sensitisation about women's health during pregnancy, childbirth, and postnatal. This has been triggered by the inadequate emotional, financial, moral, and social support men render to their spouses when their spouses need it most. Men don't want to accompany their spouses for antenatal services and during childbirth, denoting that such activities are for women. In addition, men continued to expect PPMs to fulfil their obligations, especially in house chores during pregnancy, childbirth, and postpartum. This unsupportiveness has undermined MHI access and must be addressed. This study was in line with previous research arguing that Men's poor support to their female partners in pregnancy and childbirth-related issues has been found to affect women's maternal health information-seeking behaviour.^33^ Therefore, much still needs to be done in Uganda to promote awareness of men and their involvement in maternal and child health programs to improve access to MHI and services.

In addition, the study revealed that mothers were challenged by the long queues at the health facilities where they accessed MHI, yet their husbands expected them to continue fulfilling their household duties while attending antenatal and postnatal care services. To make matters worse, mothers from far away endeavoured to arrive early enough at the health facilities to find their way back home and fulfil their obligations. However, they left late in the evening, aggravated by the late operation time of health facilities, the high number of mothers, and understaffing. This finding agrees with the assertion that the long queues attributed to the high volume of patients at health facilities following the late opening, time spent travelling to health facilities to seek services, and understaffing cause long waiting times at facilities.^34,40,54^

Mothers also reported that they fail to access MHI because of the uncooperative behaviour of health workers. Most mothers revealed that they desperately wished to approach health workers about MHI. However, the health workers’ attitude discouraged mothers from further seeking information from them. This was because, many times, the workers were harsh and not helpful. Mothers also revealed that some health workers could not comprehensively explain issues when queried, yet most information is provided in English, and many mothers are illiterate. This poses a language barrier challenge, an obstacle in delivering effective, relevant, and timely patient care.^55,56^ Sometimes, health workers openly speak about the sensitive and private conditions of mothers in the presence of everyone, yet this is embarrassing. Ultimately, mothers resort to avoiding the facilities because they feel ashamed. The findings correspond to previous studies, which revealed that health workers are abusive and disrespectful towards women in many African settings.^57^ Such unpleasantly rough behaviour limits the health information-seeking behaviour of PPMs and thus calls for special attention.^26^

Beyond addressing each challenge in isolation, the strength of the proposed Mobile Health Information and Decision Support System Architecture (MHIDSSA) lies in its ability to tackle these barriers simultaneously and holistically. This study established that impediments such as limited awareness, transport difficulties, financial instability, and inadequate spouse support are deeply interconnected, each one reinforcing the other to worsen maternal health outcomes.

By integrating multiple functional components, the information access, MamaSente, transport management, Emergency Management, spouse communication, and multilingual support, the MHIDSSA provides a unified solution that mirrors the complexity of real-world barriers. For instance, improving the transport without financial accessibility would still leave mothers stranded, well timely information without language adaptation would not ensure comprehension. Therefore, an integrated approach was indispensable.

This synthesis underscores the system's novelty: it transforms fragmented interventions into a cohesive architecture that empowers mothers through simultaneous access to information, communication, finance, and emergency support. By bridging these dimensions, the MHIDSSA demonstrates that sustainable improvement in maternal health information access in Uganda does not require single interventions but a coordinated and multidimensional technological framework.

### Study Limitations

This study was qualitative in nature and relied on a passive sampling approach involving 2 health facilities, Masafu General Hospital (rural) and Komamboga Health Centre III (urban). While this design provided rich, context-specific insights into maternal health information barriers, it limits the generalizability of the findings to all regions of Uganda.

Additionally, the study focused on mothers and health workers as primary participants and therefore did not incorporate perspectives from district health administrators and transport operators, whose inclusion might have enriched the systems requirement analysis.

Data were also collected during a specific time, which may not fully capture temporal or seasonal variations in health service access, such as rainy season transport challenges.

Finally, as with most qualitative studies, the interpretation of results was influenced by participants’ willingness to share their experiences and by the researchers’ analytical lens. These limitations, however, do not undermine the credibility of the findings; rather, they highlight the need for further quantitative validation and the pilot testing of the proposed architecture for feasibility and effectiveness in diverse Ugandan settings.

### Future Work

Building on the findings of this study under the proposed Mobile Health Information and Decision Support System Architecture (MHIDSSA), Future work will focus on developing and testing a functional prototype of the system referred to as the MamaApp. The prototype will integrate key modules such as Information Access, Mama Sente (financial transaction), Transport Management, Communication, and Emergency Management.

Subsequent phases will involve pilot implementation studies in both rural and urban settings to evaluate the system's usability, acceptability, and impact on maternal health information access and the decision-making among mothers. The feedback from the end users and health workers will be used to refine the system functionality and ensure cultural and linguistic adaptability.

Future research will also focus on integrating the MHIDSSA with existing health information systems in Uganda, such as the District Health Information System (DHIS2), to strengthen interoperability and scalability. These steps will provide a pathway towards a national digital ecosystem that supports inclusive, sustainable, and evidence-based maternal health services.

## CONCLUSION

This study identified multiple interrelated challenges, including limited access to maternal health information, financial instability, long distances to health facilities, inadequate spouse support, and language barriers, that collectively hindered timely informed maternal health decisions. Addressing these barriers requires a solution that integrates rather than isolates intervention.

In response, the proposed mobile health information and decision support system architecture (MHIDSSA) presents a unified, context-aware digital framework that connects mothers, health workers, and community actors. Its modular design, comprising Information Access, Mama Sente (financial transaction), Transport Management, Communication, Emergency Management, and Multilingual Support, ensures that informational, financial, social, and logistical needs are addressed simultaneously. This intervention distinguishes MHIDSSA from existing single-function mhealth tools.

The findings of this study have significant implications for maternal health service delivery and digital health policy in Uganda. If implemented, the MHIDSSA can substantially improve access to maternal health information, promote equal participation of women in health decision-making, and strengthen the health systems’ responsiveness to emergencies.

It is therefore recommended that pilot funding be sought to develop and test the MHIDSSA Prototype (MamaApp) within selected rural and urban sites to assess its usability, acceptability, and impact. Scaling such an evidence-based, integrated architecture could transform maternal health information access and contribute to achieving Uganda's maternal and child health targets under Sustainable Development Goal 3.
